# Biomimetic membrane nanotechnology in cerebral ischemic stroke:a promising technology for therapeutic treatment and diagnosis

**DOI:** 10.3389/fbioe.2025.1605377

**Published:** 2025-08-20

**Authors:** Yu Long, Zhiyan Zou, Yuanyuan Wu, Huiyi Feng, Ting Chen, Zhi Yang, Xuemin Jian, Yuan Yin, Xiaoan Li

**Affiliations:** 1 NHC Key Laboratory of Nuclear Technology Medical Transformation, Mianyang Central Hospital, School of Medicine, University of Electronic Science and Technology of China, Mianyang, China; 2 State Key Laboratory of Southwestern Chinese Medicine Resources, School of Pharmacy, Chengdu University of Traditional Chinese Medicine, Chengdu, China; 3 School of Animal Science, Xichang University, Xichang, China; 4 Department of Gastroenterology, National Clinical Key Specialty, Mianyang Central Hospital, School of Medicine, University of Electronic Science and Technology of China, Mianyang, China

**Keywords:** biomimetic membrane nanopreparations, cerebral ischemic stroke, therapy, diagnostics, development prospects

## Abstract

Cerebral ischemic stroke (CIS) is a severe cerebrovascular disease that poses numerous challenges in diagnosis and treatment, primarily attributed to blood-brain barrier (BBB) constraints and inherent drug targeting limitations. Biomimetic membrane nanotechnology, as an emerging therapeutic approach, offers a novel therapeutic strategy by emulating biological membrane structures and functions. This review comprehensively examines biomimetic nanomedicines (BMNPs) in CIS management, encompassing preparation methodologies, material characterization, and specific diagnostic/therapeutic applications. We discussed in detail various types of biomimetic nano-materials such as conventional extracellular membranes, bacterial outer membranes, and virus-like particles, and explore their capacity in enhancing BBB penetration, improving target specificity, and evading immune clearance. Current challenges regarding biosafety profiles, manufacturing quality control, targeted modification precision, and controlled drug release kinetics are delineated in this review. Looking to the future, advancing synergies between nanotechnology and biomedicine hold significant promise for optimizing CIS theranostics and expanding clinical treatment modalities.

## Graphical Abstract

**Figure:**
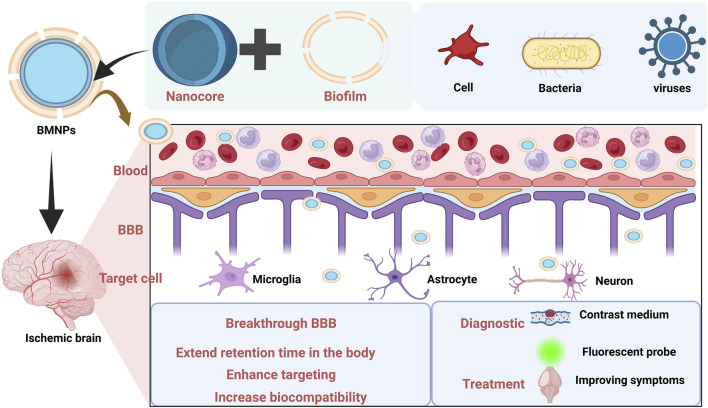

## 1 Introduction

Stroke, defined as a cerebrovascular disorder induced by cerebrovascular obstruction or rupture, manifests primarily as neurological deficits (Mohammad, 2020; Kuklina et al., 2012; Hankey, 2014). Based on pathophysiology, stroke is classified into hemorrhagic stroke and cerebral ischemic stroke (CIS), with the latter constituting the predominant subtype (accounting for about 65.3% of all acute cerebrovascular diseases) (Long et al., 2020; Maida et al., 2020). CIS is characterized by high incidence, disability and mortality rates, imposing substantial clinical and socioeconomic burdens on affected individuals and healthcare systems (Tao et al., 2020). Beyond direct cerebral injury, ischemic stroke may trigger systemic complications including multi-organ dysfunction syndrome due to neuroinflammatory cascades and hemodynamic instability.

The current guiding principle for CIS managementis rapid restoration of cerebral blood flow through timely reperfusion. Clinically validated treatments for CIS remain limited, primarily constrained to mechanical thrombectomy and intravenous thrombolysis (Weafer et al., 2019). Within the 24-h therapeutic window post-symptom onset, mechanical thrombectomy enables direct clot extraction from occluded vessels. However, its clinical adoption is hindered by specialized equipment requirements and procedural complexity (Walter, 2022). Furthermore, this intervention carries substantial surgical risks (e.g., vessel dissection, distal embolization), restricting its application to acute stroke patients with contraindications to or failure of thrombolytic therapy (Berge et al., 2021; Campbell et al., 2015). The commonly used drugs at present are second-generation tissue plasminogen activators (tPA). Although third-generation thrombolytic demonstrate improved fibrin specificity, their cost-effectiveness barriers impede widespread implementation. While tPA reduces long-term disability risk in acute CIS, it is associated with a 6% incidence of symptomatic intracranial hemorrhage.

Nano-drug delivery systems, as engineered carriers, demonstrate significant potential in enhancing the blood-brain barrier (BBB) penetration of neuroprotective agents, prolonging systemic circulation half-life, and improving bioavailability (Chen J. et al., 2022). Conventional nanocarriers include liposomes, nanoemulsions, hydrogels, and inorganic nanoparticles, which have been extensively utilized in preclinical research for targeted drug delivery (Zhao et al., 2021; Liu Y. et al., 2021). However, conventional nanocarriers face critical limitations, including inadequate targeting, premature drug leakage, poor stability and biocompatibility, and short systemic circulation half-lives. To overcome these constraints, the exploration of novel nano-drug delivery systems beyond traditional paradigms holds significant translational value (Liao et al., 2023). With the development of the biopharmaceutical field, researchers have constructed a type of biocompatible nanomaterial, known as biomimetic membrane nanopreparations (BMNPs).

Biomimetic membrane nanotechnology constitutes an interdisciplinary field that mimics the structural and functional attributes of biological systems, with a core objective of replicating the sophisticated designs observed in nature to engineer nanoscale materials with precise functionalities (Ryu et al., 2019; Arya et al., 2023; Jia et al., 2024). This approach has gained significant traction in biomedical applications, particularly in targeted drug delivery and precision therapeutics, owing to its transformative potential (Wu et al., 2022). Recent advances have spotlighted biofilm-derived biomimetic functional materials (e.g., extracellular vesicles, membrane-coated nanoparticles) for disease intervention, driving substantial research interest. Biomimetic drug delivery systems leveraging biofilm modifications offer unique advantages: they retain the drug encapsulation efficiency of nanocarriers while preserving ligands essential for cellular physiological functions. These biomimetic nanoformulations exploit the intrinsic homing capacity of membrane proteins to achieve site-specific targeting. Common membrane carriers—including erythrocytes, macrophages, platelets, tumor cells, and engineered *Escherichia coli* membranes—exhibit properties of natural delivery vectors, such as superior biocompatibility, low immunogenicity, and high binding specificity (Malhotra et al., 2022; Desai et al., 2023).

Recent advances in nanotechnology have propelled BMNPs to demonstrate transformative potential for CIS theranostics. Even though the full clinical realization of biomimetic membrane nanotechnology remains to be achieved, its evident benefits and the wide availability of membrane sources lay a strong foundation for its scalable production and integration into personalized precision medicine. This review comprehensively analyzes research progress of BMNPs on membrane source engineering and diagnostic-therapeutic integration in CIS intervention. This review aims to accelerate clinical translation of engineered BMNPs, ultimately advancing next-generation neurovascular interventions for CIS.

## 2 Delivery bottlenecks

### 2.1 Limitations of BBB

The optimization of therapeutic strategies for CIS, one of the leading global causes of mortality and disability, remains a central focus of medical research. Current treatment efficacy is suboptimal, partly attributable to multi-level physiological barriers that impede drug delivery (Al-Ahmady et al., 2019). Notably, the BBB and “no-reflow” phenomenon critically restrict drug access to deep ischemic regions, presenting major therapeutic challenges for CIS (Wang et al., 2025; Sweeney et al., 2019). Anatomically, the BBB comprises brain microvascular endothelial cells interconnected by tight junctions, surrounded by pericytes and astrocytic end-feet, all anchored within a specialized basement membrane (Langen et al., 2019). Vascular endothelial cells constitute the primary structural component of the BBB. Their membranes express efflux transporter proteins, and both elements collectively ensure the BBB’s barrier function (Takata et al., 2021). Cerebral microvascular endothelial cells form a continuous tight junction network, encircled by pericytes and embedded within a specialized basement membrane. The close arrangement and interaction of these structures form a highly selective barrier that dynamically regulates substance exchange to maintain CNS homeostasis. The BBB functions as a dynamic vascular barrier that restricts neurotoxic substances (e.g., toxins and pathogens) from entering brain tissue, thereby protecting neural homeostasis (Wang J. et al., 2024). It has been reported that 100% of large molecule drugs (>500 Da) and 98% of small molecule drugs do not cross the BBB (Xie et al., 2019). Consequently, the BBB represents the primary bottleneck for CIS pharmacotherapy.

### 2.2 Drug properties and other barriers

Due to their intrinsic properties, drugs administered into the body often exhibit a short half-life, poor targeting to lesion sites, and side effects. Research over recent decades has shown that nanocarrier systems can improve drug solubility, stability, release characteristics, safety and targeting properties. Nanocarriers demonstrate significant potential for enhancing drug delivery owing to their distinctive physicochemical properties. As a multi-target therapeutic agent for CIS, n-butylphthalide exhibits extremely low aqueous solubility and limited oral bioavailability, substantially restricting its clinical utility via oral administration. Studies reveal that sodium cholate-modified liposomes encapsulating n-butylphthalide achieve markedly superior cellular uptake and transport efficiency compared to free n-butylphthalide. Notably, this formulation yields an approximately 4-fold increase in absolute bioavailability relative to conventional n-butylphthalide suspension (Zhang et al., 2021). In rat models of middle cerebral artery occlusion (MCAO), the n-butylphthalide-loaded sodium cholate liposomes demonstrated pronounced therapeutic efficacy, indicating their potential for targeted ischemic stroke treatment.

Despite the advantages of nano-drug delivery systems, they lack intrinsic active targeting capabilities and face significant delivery limitations upon entering the body. As exogenous substances, they are recognized and phagocytosed by blood phagocytes—particularly macrophages and neutrophils—resulting in rapid clearance by the reticuloendothelial system. (Tang et al., 2019). Upon systemic administration, nanomedicines rapidly adsorb nonspecific plasma proteins to form a “protein corona”. This corona triggers rapid clearance by the reticuloendothelial system, significantly curtailing their circulation half-life. Critically, the protein corona masks surface modifications (e.g., targeting ligands), thereby ablating their intrinsic active targeting capabilities (Li H. et al., 2021). Beyond the BBB, the cell membrane barrier constitutes a major impediment to drug efficacy. The cell membrane, composed of a lipid bilayer embedded with proteins, is a physical barrier between the internal and external environments of the cell while serving protective and interactive functions. Therefore, when a drug is delivered to a target cell, its transfer to the cell is closely related to the cell membrane barrier (Hegde et al., 2022).

**FIGURE 1:**
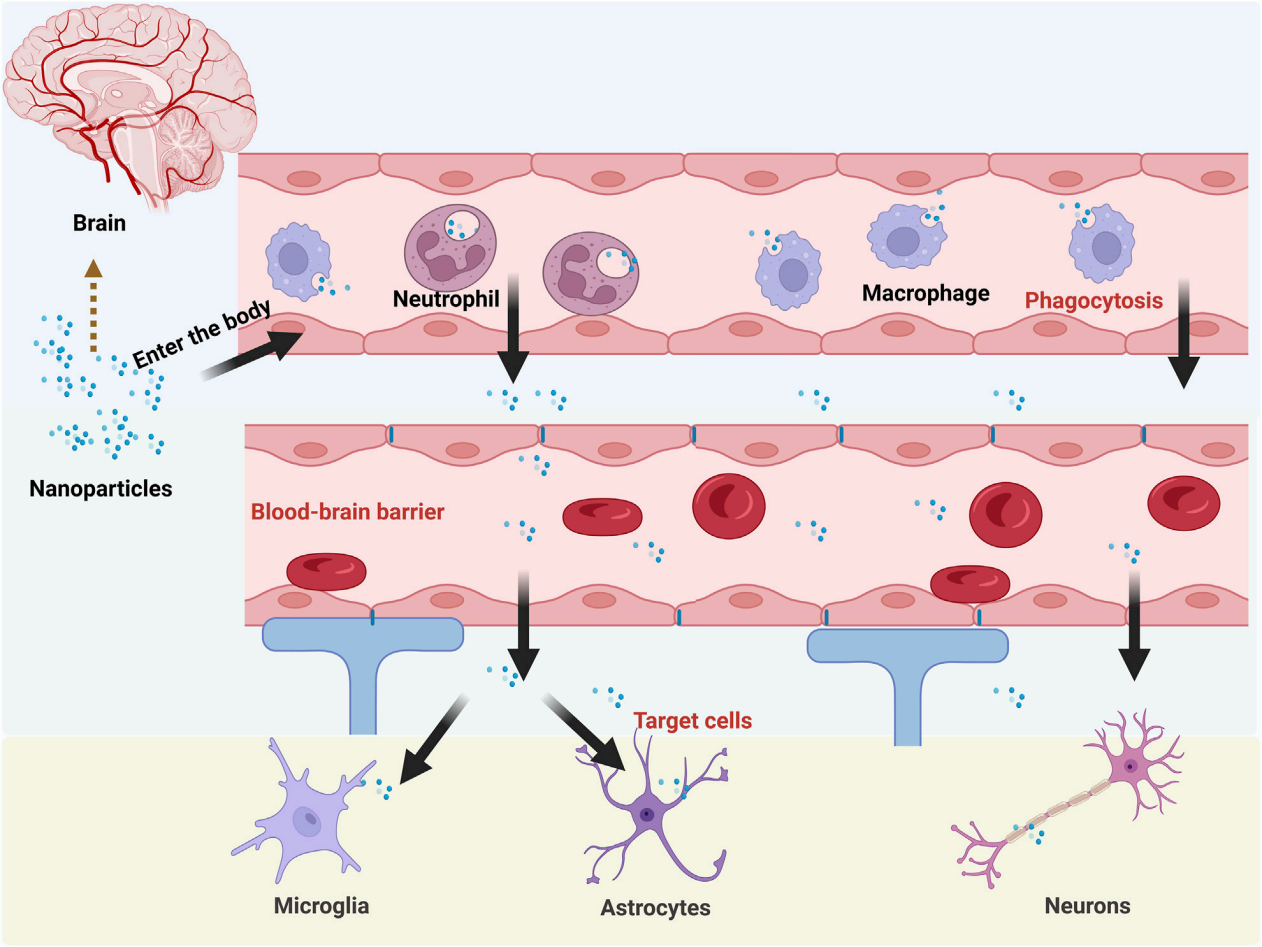
Delivery limitations faced by common nanoformulations after entering the body.

Although nanoformulations enhance drug properties, their delivery efficacy remains constrained by complex physiological environments. Compared with conventional nanocarriers, novel endogenous carriers composed of erythrocytes, platelets, macrophages, tumor cells, and bacteria exhibit superior biocompatibility and targeting specificity. However, conventional bio-carriers often struggle to penetrate and be efficiently taken up by specific tissues due to their own physicochemical properties. Meanwhile, cellular carriers are limited by the defects of low drug loading capacity and potential instability. In contrast, BMNPs rcombine the biological and targeting advantages of biofilms with the stability and high drug loading capacity of nanoformulations. As a result, BMNPs are increasingly attracting researchers’ attention for their potential applications in biomedicine. The common delivery limitations faced by nanomedicines after entering the body are shown in Figure 1.

## 3 Preparation and characterization of BMNPs

### 3.1 Preparation method

**FIGURE 2:**
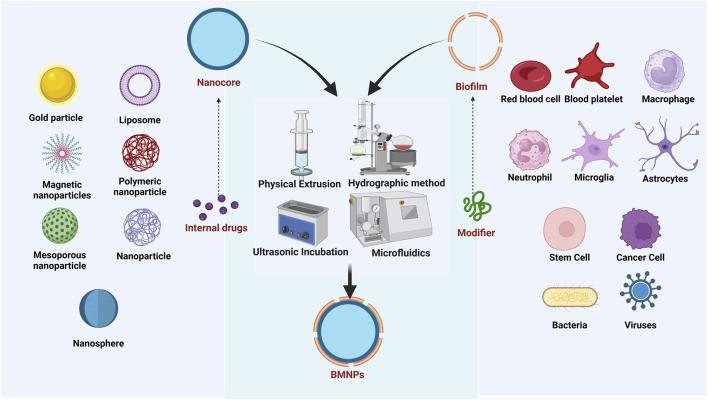
Preparation process of BMNPs.

The fabrication of BMNPs encompasses three critical stages: membrane isolation, core nanocarrier synthesis, and membrane-core fusion, as shown in Figure 2 (Wu et al., 2022). Membrane extraction protocols are tailored to the biological source type, as cells—fundamental structural and functional units of organisms—derive specialized functions primarily from their membrane-surface antigenic profiles. Consequently, preserving membrane protein integrity and preventing degradation during extraction is essential. For anucleated cells, such as mammalian mature erythrocytes and platelets, membranes are isolated via hypotonic lysis or freeze-thaw cycles, followed by differential centrifugation to remove soluble proteins and harvest membrane vesicles. For nucleated eukaryotic cells, isolation from tissues or cultured systems precedes membrane disruption through ​hypotonic treatment, sonication, or repeated freeze-thawing; organelles are then separated by differential centrifugation, and membrane fragments are ultrasonicated to form vesicles (Chen et al., 2020). Bacteria inherently comprise structural components such as peptidoglycan, lipopolysaccharide (LPS), lipoteichoic acid (corrected from “lipophosphatidic acid”), flagella, and nucleic acids (DNA/RNA). For Gram-negative bacteria like *Escherichia coli*, outer membrane vesicles can be isolated from culture supernatants through sequential centrifugation and ultrafiltration: initial low-speed centrifugation removes bacterial cells, followed by filtration to eliminate debris, and final concentration via ultrafiltration membranes to enrich outer membrane vesicles (Gao et al., 2022). Ruichi Liu obtained *Salmonella* membranes by ultrasonication followed by centrifugation (Liu R. et al., 2024). Many viral capsid proteins possess the inherent capability for reversible self-assembly, enabling them to form structurally authentic viruslike particles (VLPs) that closely mimic native viral capsids. Both native virions and VLPs are collectively termed virus-based nanoparticles (VNPs), serving as modular building blocks for constructing composite nanostructures and functional biomaterials. The production of VNPs frequently relies on precision engineering strategies—either through genetic modifications that insert functional sequences into CP genes or chemical conjugations that attach therapeutic payloads to coat protein residues. These engineered nanoparticles are generated using diverse systems including bacterial, insect, yeast, and plant cell cultures via fermentation or bioreactor processes, as well as cell-free expression platforms that expedite rapid prototyping (Arul et al., 2024). Current manufacturing platforms enable high-fidelity, large-scale production of VNPs and their structural components via cellular expression systems (including native or recombinant hosts). Enveloped VNPs are typically generated as replication-competent virions through *de novo* assembly within host cells. In contrast, non-enveloped VNPs exhibit versatile assembly pathways—undergoing intracellular maturation into preformed capsids or extracellular self-assembly from purified coat protein subunits post-cell lysis.

The inner core nanomaterials serve as foundational components in BMNPs. In recent years, various types of nanomaterials, mainly categorized as organic nanoparticles and inorganic nanocrystals, such as polymer nanoparticles, liposomes, silica, mesoporous silica, gold particles, iron oxide, metal-organic skeletons, nanogels and black phosphorus have become the core carriers in cell membrane biomimetic technology. These materials are extensively employed for nanoparticle fabrication owing to their excellent biocompatibility and exceptional drug-loading capacity. BMNPs are synthesized by a variety of methods, including physical extrusion, hydropathic methods, ultrasonic incubation, and microfluidic techniques (Bao et al., 2023) (Table 1). Each fusion methodology for BMNPs presents distinct advantages and limitations, with physical extrusion and ultrasonic incubation emerging as the most prevalent techniques. These approaches enable the integration of natural biofilms with synthetic nanocarrier cores, yielding novel biomimetic drug delivery systems characterized by prolonged *in vivo* circulation half-life, enhanced biocompatibility, and superior targeting specificity.

**TABLE 1:** Preparation methods of BMPNs.

Methods	Biofilm-nanocore	Advantages	Disadvantages	References
Physical extrusion	Macrophage membrane protein- lincRNA-EPS-liposome *Escherichia coli* Outer Membrane Vesicles - Gold Nanocages Macrophage membrane-baicalein liposomes Attenuated *Salmonella* bacterial outer membrane vesicle-Pluronic (Pluronic) F127	Adjustable particle size of BMNPs by pore size of the filter membrane; convenient operation; good reproducibility	Easily intercepted biofilm on the filter membrane, resulting in losses; not suitable for large-scale production	Zhang et al. (2020), Zafar et al. (2024), Long et al. (2022), Chen et al. (2017)
Hydrographic method	Tumor cell membrane-paclitaxel liposomes	The properties of the liposomes formed are stable and more structurally complete	Organic solvent residue; low encapsulation rate; uneven particle size distribution; not applicable to all types of nanopreparations; relatively low production efficiency, not suitable for large-scale production	Li (2019)
Ultrasonic incubation	Red blood cell membrane-Ru- Se@GNP; macrophage membrane-cloaked liposome loaded with Ginsenoside Rg3 and Panax notoginseng saponins *Escherichia coli* outer membrane vesicles -Gold nanoparticles; Salmonella-Gold nanorods	Simple operation; saving raw materials; facilitating film coating; applicable to a wide range of nanocarriers	Poor particle homogeneity; uneven film coating; localized high temperature effects; unsuitable for heat-sensitive substances	Gao et al. (2022), Liu R. et al. (2024), Liu T et al. (2024), Lin et al. (2019)
Microfluidics	Erythrocyte membrane-derived vesicle-Fe_3_O_4_ magnetic nanoparticles	Precise control of particle size and distribution; high throughput and continuous production; good repeatability and stability; simple operation	High equipment cost; high technology threshold; complicated cleaning and maintenance; limited scope of application; production scale limitation	Rao et al. (2017)

The physical extrusion method generally utilizes mechanical force to fuse nanomaterials with membranes, offering procedural simplicity but limited scalability.

However, significant material loss occurs as phospholipids and membrane proteins adsorb onto polycarbonate membranes during hybrid vesicle preparation, compromising cellular uptake efficiency. In contrast, the ultrasonic incubation method circumvents these material losses yet imposes stringent core mechanical strength requirements, precluding its use for fragile biomimetic drug carriers.

### 3.2 Evaluation of properties

#### 3.2.1 Physical and chemical properties

Post-preparation characterization of BMNPs requires assessment of fundamental parameters—including particle size, zeta potential, and morphology—to optimize formulation outcomes. Achieving monodisperse nanoparticles with controlled dimensions (typically 100–200 nm for BMNPs) is critical to minimize aggregation and ensure pharmaceutical efficacy, safety, and stability. Consequently, particle size distribution represents the paramount quality attribute for nanomedicines. Zeta potential measurements elucidate surface charge alterations induced by biomembrane coating. Dynamic light scattering serves as the primary technique for determining nanoparticle size distribution and zeta potential. Notably, BMNPs generally exhibit larger hydrodynamic diameters than their unmodified counterparts. Surface charge governs physiological stability and protein corona formation; the characteristic negative charge of biomembranes typically increases nanoparticle electronegativity post-encapsulation. This enhanced negative charge may improve colloidal stability by electrostatic repulsion and reduce interparticle aggregation. Such physicochemical transformations may further modulate biodistribution patterns. Morphological validation via transmission/scanning electron microscopy confirms core-shell architecture and membrane integrity. Negative-stained transmission electron microscopyreveals nanoparticle topology consistent with the native nanocarrier structure. Drug loading capacity, encapsulation efficiency, and *in vitro* release kinetics of BMNPs are evaluated using methods tailored to the nanocarrier and drug properties (e.g., emulsion destabilization, ultrafiltration centrifugation, dialysis). These optimized physicochemical properties confer unique advantages for targeted drug delivery, enhancing therapeutic efficacy while mitigating systemic toxicity.

#### 3.2.2 Biological properties

Biomembrane fragments retain native biological constituents and membrane protein profiles post-isolation. BMNPs consequently inherit targeting capability and biocompatibility while preserving core nanoparticle functionalities. Membrane proteins, particularly on cell-derived biomembranes, dictate biological performance and determine whether BMNPs maintain source-cell properties. Contrary to assumptions of perfect fusion, Lizhi Liu et al. demonstrated via fluorescence burst assay that up to 90% of BMNPs exhibit partial coating. Notably, suboptimally coated nanoparticles remain internalizable by target cells. Integrated molecular dynamics and experimental analyses revealed that high-coverage nanoparticles (≥50%) undergo individual cellular uptake and low-coverage nanoparticles (<50%) require aggregation prior to internalization (Liu L. et al., 2021).

Functional validation of membrane bioactivity is essential for therapeutic efficacy. Transmission electron microscopy and particle sizing can be initially used to verify successful encapsulation, evidenced by significant particle diameter enlargement post-modification. Currently, protein bands from isolated biofilms, unencapsulated nanoparticles, and membrane-encapsulated nanoparticles can be detected by gel electrophoresis, and the protein composition of the membranes can be analyzed to verify the integrity of the membrane surface proteins and membrane signature proteins. Complementarily, contemporary studies implement dual-fluorescence labeling techniques where distinct fluorophores tag membranes and nanocarriers. Laser scanning confocal microscopy visualizes membrane-nanoparticle co-localization, while flow cytometry quantitatively measures target cell uptake efficiency of the resultant biomimetic nanoparticles. However, existing methods could verify protein composition but cannot detect post-encapsulation membrane structural compromises.

## 4 Application of BMNPs in the treatment of CIS

### 4.1 Conventional extracellular membranes

Red blood cells, a type of cell in the blood, whose main function is to carry oxygen and carbon dioxide. Red blood cell membrane-modified nanoparticles harness specific receptors and ligands on the erythrocyte surface to enhance BBB penetration, thereby promoting drug delivery efficiency to brain ischemic regions. Yinli Cao engineered reactive oxygen species (ROS)-responsive nanocarriers with red blood cells membranes functionalized by stroke-homing peptide (SHp) for targeted rapamycin delivery, prolonging circulation and enhancing ischemic targeting (Cao et al., 2024). Similarly, a similar bioengineered nanocarrier for stroke-specific delivery of the neuroprotective agent NR2B9C was developed by Wei Lv et al. The nanocarrier consisted of a dextran polymer core modified with ROS-responsive boronic ester and red blood cell membrane shell inserted with SHp (Lv et al., 2018).

Platelet aggregation, a primary trigger of thrombotic disorders, represents a key therapeutic target for CIS. Consequently, platelet membrane-based biomimetic nanoparticles demonstrate significant potential for site-specific drug delivery to ischemic lesions and atherosclerotic plaques. Junyan Song designed sialic acid-modified platelet membrane-coated Prussian blue nanozymes loaded with deoxyribonuclease I, enabling neutrophil-mediated BBB penetration to alleviate oxidative stress (Song et al., 2024). Ruibing Wang et al. developed a tissue plasminogen activator (tPA) delivery system based on AnnexinV and platelet membranes to improve the targeting efficiency of tPA to the site of thrombosis and to enhance thrombolysis through the dual action of platelet membranes and Annexin V (Quan et al., 2022). Mingxi Li et al. engineered platelet membrane-coated magnetic nanoparticles (PAMNs) co-loading L-arginine and γ-Fe_2_O_3_. Leveraging specific membrane proteins and external magnetic guidance, this system enables rapid *in vivo* accumulation and in situ nitric oxide (NO) generation to promote vasodilation and blood flow restoration, thereby extending the CIS therapeutic window (Li et al., 2020).

Jianglong Kong et al. engineered platelet membrane-encapsulated Cu_4_._6_O nanoparticles for targeted codelivery of docosahexaenoic acid and recombinant human tPA, demonstrating potent thrombus-specific homing (Kong et al., 2024). Wenyan Yu et al. constructed platelet membrane-camouflaged melanin nanovesicles co-loaded with tPA. This platform integrates platelet-mediated thrombus targeting, melanin-driven photothermal conversion, and intrinsic free radical scavenging for cascade-enhanced CIS therapy (Yu et al., 2022). Qinghua Wu et al. synthesized dual-membrane (platelet/microglia) hybrid nanoparticles with inflammation-directed ROS responsiveness. By combining thrombus targeting (platelet membrane) and microglial isoform specificity, this system enables efficient *in vivo* delivery of siSPHK-1 to silence genes in microglia while reprogramming the inflammatory microenvironment at CIR injury sites (Wu, 2023).

Cancer cell membrane-endothelial receptor interactions critically mediate BBB transmigration during metastasis (Hao et al., 2023). Zhang et al. engineered 4T1 cell membrane-camouflaged pH-sensitive nanocarriers (MPP/SCB) co-loading succinylphenol. This biomimetic platform exploits 4T1-derived BBB penetrability to achieve targeted delivery to cerebral ischemic lesions, significantly attenuating ischemia-reperfusion injury (He et al., 2021). Lu Tang et al. developed liposomes co-loaded with salvinorin/polymetformin, cloaked by hybrid 4T1 tumor cells and platelet membranes. By synchronizing 4T1-mediated BBB trafficking with platelet-specific vascular damage targeting, this system spatiotemporally reduced neuronal apoptosis, infarct volume, and restored neurological functions (Tang et al., 2024).

Neutrophils, the predominant leukocyte population in human blood, utilize amoeboid motility and phagocytic capacity to eliminate pathogens via granular enzymes (e.g., myeloperoxidase, lysozyme). Their unique expression of integrin β2, macrophage-1 antigen, and lymphocyte function-associated antigen 1 enables rapid recruitment to inflamed brain microvascular endothelia. Leveraging this intrinsic homing mechanism, Lishuai Feng et al. engineered neutrophil membrane-camouflaged mesoporous Prussian blue nanozymes (MPBzyme@NCMs) for active ischemic stroke targeting, significantly enhancing nanozyme delivery to ischemic brain regions through receptor-mediated tropism (Feng et al., 2021). While fingolimod hydrochloride (FTY720) demonstrates neuroprotective potential in ischemic brain parenchyma, its systemic delivery is limited by poor BBB penetration and cardiotoxicity risks. Ya Zhao et al. engineered ROS-responsive prodrug nanoparticles camouflaged by differentiated HL-60 cell membranes, enabling targeted FTY720 release in ischemic regions. This design minimizes off-target toxicity while suppressing infection risks (Zhao et al., 2024). Zhuang Tang et al. engineered Leo@NM-Lipo nanovesicles merging neutrophil membranes with leonurine-loaded liposomes. The platform concurrently enhanced BBB penetration/reparation and remodeled the post-ischemic microenvironment to reverse I/R injury (Tang et al., 2023). Concerned about the neutrophil membrane’s tropism for the infarct core and affinity for inflammatory cytokines, Shanshan Liu et al. constructed neutrophil membrane-coated poly (lactic-co-glycolic acid) nanoparticles decorated with α-lipoic acid (NM/PLGA@LA), which could neutralize inflammatory mediators in the penumbra to rescue neurons through infarct-core tropism and cytokine affinity (Liu et al., 2022). Zhufeng Dong et al. Developed SHp-modified, neutrophil membrane-encapsulated nanoparticles for ROS-triggered edaravone delivery. This system demonstrates 5.16-fold enhanced targeting specificity toward inflammatory microenvironments vs. non-camouflaged counterparts (Dong Z. et al., 2024).

Macrophages, ubiquitous mononuclear phagocytes, could orchestrate immunomodulation, phagocytosis, and antigen presentation. Jie Ma et al. engineered macrophage membrane-camouflaged nanocomplexes (AK137/fibronectin) co-delivering edaravone. This biomimetic system traverses the BBB via macrophage membrane, reprogramming microglia to restore cerebral hemodynamics post-CIR injury through vascular regeneration (Ma J. et al., 2024). Apelin-13 can effectively protect neurons from CIS injury, and it has been shown that by using macrophage membranes coated with distearoyl phosphatidylethanolamine-polyethylene glycol-RVG29 peptide, which effectively crosses the BBB, Apelin-13 could be effectively delivered to the inflammatory regions (Ma C-S. et al., 2024). Tianshu Liu et al. developed Ginsenoside Rg3 and Panax notoginseng saponins-loaded liposomes cloaked by macrophage membranes. These vesicles attenuate neuroinflammation and infarct volume while enhancing BBB penetration (Liu T. et al., 2024). Chao Li et al. designed macrophage membrane-coated MnO_2_ nanoparticles encapsulating fingolimod. These nanoparticles actively accumulate in the damaged brain through macrophage membrane protein-mediated recognition of cell adhesion molecules overexpressed on the damaged vascular endothelium (Li C. et al., 2021). Beyond conventional drug delivery, macrophage membrane proteins enable targeted *in vivo* transport of engineered long intergenic non-coding RNAs (lincRNAs). Benping Zhang et al. formulated macrophage membrane-protein-modified liposomes encapsulating artificial lincRNA-EPS. These nanovesicles achieve immune evasion and inflammatory-site homing in brain tissues, releasing lincRNA-EPS to suppress neuroinflammation while promoting neuronal regeneration (Zhang et al., 2020). Activated macrophages polarize into pro-inflammatory M1 and anti-inflammatory M2 phenotypes. M2 macrophages exert neuroprotective effects through secretion of anti-inflammatory cytokines and efferocytosis (phagocytic clearance of cellular debris), with their membranes demonstrating constitutive capacity to neutralize pro-inflammatory cytokine (Long et al., 2024; Nakkala et al., 2022). Shanshan Zhang et al. engineered M2 macrophage membrane-camouflaged poly (lactic acid) -hydroxyacetic acid copolymer acid nanoparticles loaded with baicalin. The M2-derived membrane components neutralize pro-inflammatory cytokines, enabling targeted accumulation in ischemic brain microglia and neurons (Zhang et al., 2025). Monocytes, a type of white blood cell, are part of the immune system and are the largest blood cells in the bloodstream with a variety of functions, including defense against infection, removal of dead cells and tissue debris, and participation in inflammatory responses. Yanyun Wang et al. developed monocyte membrane-coated rapamycin nanoparticles that attenuate CIS injury by inhibiting monocyte infiltration and microglial proliferation (Wang et al., 2021).

Microglia, the resident immune cells of the brain, serve as primary responders in CIS pathology and key regulators of neuroinflammation. Ruiqi Cheng et al. engineered microglia membrane-camouflaged nanoparticles conjugated with anti-rejection guidance molecule a (anti-RGMa) monoclonal antibodies. This biomimetic design leverages endogenous microglial tropism to target ischemic-damaged endothelial cells following intravenous administration (Cheng et al., 2024). Similar to macrophages, microglia polarize into distinct pro-inflammatory (M1) and anti-inflammatory (M2) phenotypes in response to pathological stimuli (Long et al., 2024). Leveraging the inherent ischemic homing and BBB penetrability of M2 microglia membranes, Ranran Duan et al. engineered a biomimetic nanoscavenger wherein peroxidase-encapsulated tannic acid nanoparticles were camouflaged with M2 membranes for targeted CIS therapy (Duan et al., 2022). Concurrently, Wenxiu He et al.constructed an oxygen-delivering nanoplatform utilizing M2 microglia membrane-modified PEG-SMA-F11/P123 nanoparticle; this system capitalizes on the high affinity of M2 membranes for inflamed cerebrovascular endothelia to augment penumbral oxygen partial pressure during early ischemia, suppressing apoptosis, and inhibit matrix metalloproteinase-9 (MMP-9) secretion, thereby preserving BBB integrity and attenuating cerebral edema (He et al., 2023).

Astrocytes serve as pivotal modulators in CIS, critically influencing neuronal injury, neuroinflammation, and cellular immunity (Jadhav et al., 2023). Leveraging their innate biomimetic properties—including immune evasion and isotype targeting—Zihao Liu engineered CXCR3 receptor-modified astrocyte membrane-fused nanovesicles co-delivering rapamycin and endothelin-1-targeting siRNA, where membrane surface engineering significantly enhanced nanovesicle homing to infarcted lesions (Liu Z. et al., 2024).

Stem cell therapy offers regenerative potential for ischemic stroke through cellular differentiation and tissue repair (Ya et al., 2024), yet intravenous transplantation faces challenges of low targeting efficacy. Stem cell membranes have emerged as promising targeting vectors, leveraging their intrinsic homing capacity for diseased tissues. Qi Zhang et al. engineered mesenchymal stem cell membrane-camouflaged Prussian blue nanoparticles loaded with ZL006, significantly reducing immunogenicity while enhancing active homing to ischemic penumbrae (Zhang et al., 2024). Similarly, Yun-Fei Dong et al. utilized mesenchymal stem cell membrane-modified liposomes for targeted Dl-3-n-butylphthalide delivery, improving cerebral microenvironments and attenuating neuroinflammation to restore motor function (Dong Y-F. et al., 2024). Jinjin Shi et al. further constructed CXCR4-overexpressing mesenchymal stem cell membrane-coated nanoparticles encapsulating A151, which could adsorb and neutralize CXCL12 to block peripheral neutrophil/macrophage infiltration while normalizing immune microenvironments (Shi et al., 2022). The therapeutic CIS effects of BMNPs prepared from cell membrane sources are shown in Supplementary Table S2.

### 4.2 Bacterial outer membrane

Bacterium-derived drug delivery systems, leveraging advancements in biochemical and genetic engineering, have emerged as pivotal biomedical tools for CIS intervention. These platforms overcome physiological barriers, including the BBB, to actively target inflammatory microenvironments. Similar to cytomembrane-based bionanomic systems, bacterial outer membranes are integrated with functionalized nanoparticles for diversified therapeutic delivery. (Hosseinidoust et al., 2016). Notably, Gram-negative bacteria naturally secrete outer membrane vesicles (30–200 nm diameter) containing pathogen-associated molecular patterns such as lipopolysaccharides outer membrane proteins, and lipoproteins. These outer membrane vesicles function as potent immunomodulators, triggering inflammatory responses that enhance innate immunity while serving as versatile vaccine adjuvants for both humoral and cellular immune activation (Qin et al., 2022).

Shaobing Zhou demonstrated that bacterial outer membrane vesicles enhance pioglitazone delivery to the brain via lipopolysaccharide-Toll-like receptor 4 binding on neutrophils. This system inhibits NOD-like receptor family pyrin domain containing 3 inflammatory vesicles activation and ferroptosis, thereby attenuating reperfusion injury and eliciting neuroprotection (Pan et al., 2023). Complementarily, *Escherichia coli* K1 exploits outer membrane functional proteins to initiate trans-endothelial transport through receptor interactions with brain microvascular endothelial cells. Leveraging this mechanism, Han Liang et al. constructed an endotoxin-depleted *Escherichia coli* K1 bacterial outer membrane-coated nanoplatform. The biomimetic design mediates targeted brain delivery by leveraging bacterial protein-endothelial receptor interactions, enabling BBB traversal and interstitial distribution within cerebral parenchyma (Chen H. et al., 2022).

### 4.3 Virus-like particles

VNPs exhibit unique immunogenicity, enhanced biocompatibility, and host-cell invasion capabilities, positioning them as versatile nanocarriers. VLPs—engineered from viral scaffolds—integrate advantages of viral and non-viral delivery systems. Their exceptional physicochemical properties enable broad applications in materials science and biomedicine. Structurally, VNPs permit precise modification of capsid domains and genetic elements through chemical conjugation or genetic engineering. While VNP-based biomimetic systems are extensively investigated in oncology, their potential for CIS therapy remains promising yet underexplored.

Current strategies augment the RNA-loading capacity of leukocyte-derived extracellular vesicles by leveraging endogenous retrovirus-like protein capsids native to the human brain. Specifically, integrating Arc protein capsids with their cognate 5′-untranslated regions markedly enhances RNA encapsulation and delivery efficiency. This hybrid system, functionalized with endogenous envelope proteins, achieves BBB penetration for precise delivery to neuroinflammatory lesions (Gu W. et al., 2024).

## 5 Application of BMNPs in the diagnosis of CIS

Recent years have witnessed remarkable progress in BMNPs for diagnostic imaging of IS. By leveraging natural membrane proteins, these bionanoplatforms enable targeted accumulation, immune evasion, and prolonged circulation—collectively enhancing imaging contrast and lesion specificity. Clinically, magnetic resonance imaging (MRI) is widely utilized due to its high sensitivity and multi-sequence scanning capabilities. Iron oxide nanoparticles, recognized for their superparamagnetism, favorable biocompatibility, and relatively straightforward synthesis, have extensive biomedical applications. As MRI contrast agents, iron oxide nanoparticles provide high-contrast imaging signals. Mingxi Li et al. developed a platelet-membrane-coated biomimetic magnetic nanocarrier loaded with arginine and γ-Fe_2_O_3_ magnetic nanoparticles to delineate vascular injury networks in acute ischemic stroke. This platelet membrane functionalization enables targeted delivery and accumulation of the nanocarriers within ischemic core regions, significantly enhancing lesion classification accuracy and spatial localization precision in MRI diagnostics for both pathological assessment and injury zone mapping (Li et al., 2020). The platelet - membrane - mimetic nanoparticles synthesized by Tang and colleagues are loaded with a selective spleen tyrosine kinase inhibitor (resveratrol) and a T2 contrast agent, superparamagnetic iron oxide nanoparticles. These nanoparticles allow for real - time monitoring of thrombolysis via MRI imaging. The platelet - membrane coating enables the successful identification and adhesion of neutrophils, thereby facilitating targeted labeling and imaging of thrombi (Lin et al., 2022). MRI imaging is also frequently used to investigate drug efficacy. Zhou Shaobing et al. proposed a strategy based on outer membrane vesicles derived from the outer membrane of *Escherichia coli* “hitchhiking” on neutrophils to enhance the brain delivery of pioglitazone for treating ischemic stroke. MRI results showed that the infarct volume in the normal saline group was 46%, whereas biomimetic nanosolutions of pioglitazone group reduced the cerebral infarct volume to 14% (Pan et al., 2023).

Computed Tomography (CT) is a pivotal tool for diagnosing ischemic stroke, yet its sensitivity in detecting early-stage cerebral infarcts remains suboptimal. In CT imaging, nanoparticles have demonstrated significant potential for optimizing Micro-CT diagnostics through prolonged circulation half-lives. By refining traditional contrast agents and radiotracers, nanoparticles can enhance imaging diagnostics for CIS, serving directly as contrast agents or labeled probes to improve CT/positron emission tomography (PET) imaging accuracy (Lin et al., 2022). Currently, research on BMNPs for CT/PET imaging augmentation is limited. Theoretically, BMNPs can function as contrast agent carriers that actively target ischemic brain lesions. This targeted delivery elevates local contrast agent concentration, thereby enhancing image contrast and resolution. Such advancements are critical for precise detection and localization of pathologies like ischemic foci, vascular stenosis, or occlusions—particularly for early infarct identification—effectively addressing the diagnostic limitations of conventional CT/PET modalities.

As non - invasive or minimally invasive imaging techniques, ultrasound imaging and photoacoustic imaging play a crucial role in the early diagnosis, condition assessment, treatment guidance, and prognosis monitoring of ischemic stroke. Ruiqi Cheng engineered a microglial membrane-coated magnetic iron oxide nanoplatform encapsulating anti-repulsive guidance molecule alpha (anti-RGMa) monoclonal antibodies. This system achieves thrombus dissolution through tripartite mechanisms: low-intensity focused ultrasound-induced liquid-gas phase transition of perfluorohexane, inertial cavitation effects, and magnetophoretic propulsion of Fe_3_O_4_ nanoparticles under external magnetic guidance. For extracranial arterial thrombi, the platform enables real-time thrombus visualization via ultrasound/photoacoustic dual-modality imaging, with *in vivo* studies confirming its biosafety. Collectively, this nanoplatform exemplifies a comprehensive therapeutic strategy for ischemic stroke integrating targeted drug delivery, vascular reperfusion, and neuroprotection (Cheng et al., 2024). Leveraging the specific affinity between platelet membranes and stroke thrombi, Mingxi Li et al. platelet membranes’ inherent thrombus affinity to develop biomimetic nanobubbles that progressively accumulate and undergo coalescence within ischemic lesions, generating time-dependent ultrasound contrast enhancement signals with significant potential for auxiliary stroke diagnosis (Li et al., 2018).

Biomolecular detection represents a critical application of nanotechnology in disease diagnostics, leveraging nanoscale probes engineered to identify regulatory, signaling, and recognition biomolecules, including proteins, nucleic acids, polysaccharides, and metabolites, that serve as physiological status indicators. These nanoprobes exhibit superior sensitivity, specificity, and stability, enabling early disease detection essential for precision medicine. Biomimetic nanomaterials further enhance detection capabilities by integrating functionalized nanoplatforms with bioactive components that specifically target pathological sites with advantages including substantial surface area accommodating high-density molecular conjugation, structural specificity enabling disease-targeted imaging through surface engineering, and stimulus-responsive drug release minimizing off-target effects to optimize therapeutic outcomes (Gu Y. et al., 2024). Exemplifying this, Xiaohu Yang et al. developed a NIR-IIb nanoprobe (V&C/PbS@Ag_2_Se) for ultrasensitive *in vivo* detection of early cerebral ischemia in photothrombotic models. Intravenously administered probes targeted VCAM-1 receptors on inflamed endothelium, accumulating selectively in ischemic lesions where peroxynitrite (ONOO^−^) activated Cy7.5 oxidation to generate quantifiable NIR-IIb signals and illuminating the lesion area for highly sensitive detection (Yang et al., 2021). The pH value is an important parameter in pathophysiology. In recent years, the impact of pH changes in biological activities on normal brain function has increasingly attracted the attention of scientists. However, the reported pH sensors currently have poor biocompatibility and are limited in vivo brain detection. Concurrently, Bing Wang’s team addressed biocompatibility limitations in brain pH sensing by engineering erythrocyte membrane-cloaked tungsten microsensors. This biomimetic design circumvented immune exclusion (mitigating hyperacute/chronic rejection) while demonstrating significantly reduced neurotoxicity versus uncoated sensors during *in vivo* ischemia monitoring, establishing cell membrane modification as a critical strategy for enhancing cerebral biosensor biocompatibility (Gu Y. et al., 2024). Collectively, these advances foreshadow deeper integration of BMNPs with next-generation pathological detection technologies (Wang et al., 2019). Collectively, these advances foreshadow deeper integration of BMNPs with next-generation pathological detection technologies.

## 6 Challenges and perspectives for BMNPs

### 6.1 Safety of BMNPs

#### 6.1.1 Production and quality control

The preparation of BMNPs faces standardization challenges due to methodological variability, where divergent fabrication protocols critically impact key physicochemical properties such as size distribution, morphological integrity, and functional consistency—ultimately complicating large-scale production. The extraction and preparation of biofilm is a critical step, involving the culture collection of cells, bacteria and viruses, where the steps may have an impact on the quantity and purity quality of biofilm and its surface protein production. BMNP preparation processes exhibit inherent complexity and cost-intensiveness, further compounded by limitations in conventional monitoring methodologies. Simultaneously, the scarcity of internationally approved high-grade excipients suitable for nanopharmaceutical formulations constrains widespread clinical adoption. Crucially, maintaining structural integrity during long-term storage poses multifaceted challenges: preservation of non-degraded membrane-surface proteins demands stringent environmental controls, while stability of bionanostructures necessitates specialized transportation regimens. Translating laboratory innovations to clinical practice thus requires establishing standardized protocols—encompassing membrane extraction methodologies, comprehensive characterization of biomimetic properties, and controlled fabrication of composite nanoformulations—with particular emphasis on ensuring batch-to-batch consistency throughout manufacturing and distribution cycles. Consequently, developing rigorous quality control frameworks addressing nanomaterial homogeneity remains an urgent imperative, especially given the nascent development of regulatory policies governing BMNP standardization.

#### 6.1.2 Biological safety

Recent years have witnessed growing interest in BMNPs for *in vivo* drug delivery. Surface coat of nanoparticles with biomembrane enables biomimetic camouflage while conferring inherent biological functionalities. Compared with conventional nanoparticles, BMNPs exhibit innate targeting capabilities along with significantly reduced immunogenicity and enhanced biocompatibility. Notably, safety profiles of drugs encapsulated in biomimetic nanosystems show marked discrepancies from those of small-molecule therapeutics, suggesting fundamental variations in toxicological mechanisms that may consequently alter clinical adverse reaction profiles (Ahmad et al., 2022). Although numerous studies demonstrate the favorable biocompatibility and low toxicity of BMNPs toward healthy organs, tissues, and cells *in vivo*, comprehensive assessment of their systemic biosafety remains imperative. Potential BMNP toxicity may originate from intrinsic properties of both membrane coatings and core materials. Crucially, membranes derived from diverse biological sources exhibit distinct immunogenicity profiles, eliciting varied immune responses. Common membrane types employed for drug delivery, including erythrocyte, mesenchymal stem cell, platelet, and macrophage membranes, each carry unique immunological implications. Notably, even autologous cellular therapies require immunosuppressive regimens to prevent host rejection. The journal Cell published the results of an autotransplantation study in which the patient’s own fat cells were reprogrammed into induced pluripotent stem cells, which were then converted into pancreatic beta-like cells and transplanted to the patient. Immunosuppressive drugs are still given to prevent the body from rejecting the transplanted cells (Wang S. et al., 2024). Notably, red blood cell membrane-derived BMNPs carry hemolytic risks due to potential blood mismatches, which can trigger host immune activation via complement-mediated pathways (Delaney et al., 2016; Chen et al., 2016). Even in the presence of normal cell membranes, BMNPs administration frequently induces significant hepatic and splenic accumulation, leading to the generation of large numbers of macrophages in these organs, and phagocytosis by macrophages encourages further accumulation of nanomedicines in the liver and spleen (Zhuang et al., 2020; Wu et al., 2019). Consequently, comprehensive biosafety assessment of BMNPs impacts on healthy organs remains essential. And the inherent toxicity of nanomaterials is mainly reflected in the potential biotoxicity of different materials. *In vivo* different nanocores show different degrees of biocompatibility, and the toxicity assessment of their metabolism and degradation products *in vivo* is an important part of safety studies, which may have potential adverse reactions. Consequently, both *in vitro* and *in vivo* experiments are necessary to conduct acute and long-term toxicity evaluations.

### 6.2 Targeting modifications and drug release properties

Numerous BMNPs can traverse the BBB due to their inherent properties. However, even after successful BBB penetration, BMNPs require precise targeting to damaged brain regions or specific cell types to minimize potential toxic side effects from drug accumulation in healthy tissues and cells. Therefore, enhancing the targeting specificity of BMNPs remains a key focus of current research. Numerous current studies involve membrane engineering techniques such as hybrid biofilm formation, ligand functionalization, Polyethylene glycolylation, or glycosylation to enhance the targeting capability or stability of BMNPs(Table 2).

**TABLE 2:** Membrane engineering techniques to improve the targeting and stability of BMNPs.

Membrane engineering technologies	Membrane sources	Modification strategies	Advantages	Pharmacological effect	References
Membrane hybridization technique	Platelet-microglia	Fusion of platelet membranes and microglia cell membranes for encapsulating liposomes	Platelet membrane: thrombus-targeted properties; microglia membrane: homotypic targeting, CD29-mediated CD106 recognition, receptor-mediated endocytosis	Breaking through the *in vivo* delivery barrier of siSPHK-1, improving the inflammatory microenvironment at the site of CIR damage	Wu (2023)
	4T1 tumor cell -platelet	Fusion of 4T1 tumor cell membranes with platelet membranes for encapsulating liposomes	4T1 tumor cells: BBB penetration Platelets: targeting injured vascular system	Efficiently targeting ischemic lesion, preventing neuroinflammation, scavenging excess ROS, reprogramming microglia phenotypes, and promoting angiogenesis	Tang et al. (2024)
Ligand functionalization	Red blood cell	SHp	Targeting ischemic areas	Promoting microglia polarization, maintaining the integrity of the BBB, reducing cerebral infarction, and promoting cerebral neurovascular remodeling	Cao et al. (2024)
	Red blood cell	SHp	Targeting ischemic areas	Great protection against glutamate-induced PC-12 cytotoxicity, prolonging the somatic circulation of NR2B9C, enhancing active targeting of ischemic regions and reducing ischemic brain damage in MCAO rats	Lv et al. (2018)
	Platelet	Annexin V	Damaging phosphatidylserine to inhibit thrombosis	Improving the ability of tPA to target thrombus sites and anti-platelet activation, improving neurological function, increasing focal cerebral perfusion, reducing infarct size, and decreasing blood-brain barrier permeability	Quan et al. (2022)
	Neutrophil	SHp	Targeting ischemic areas	Precise Targeting of CIR sites, scavenging the ROS, inhibiting inflammation, and providing neuroprotection	Dong Z. et al. (2024)
	Macrophage	Distearoyl phosphatidylethanolamine-polyethylene glycol-RVG29	Transporting drugs across the BBB	Improving neurological scores and reducing infarct volume, inhibiting NLRP3 inflammasome-mediated pyroptosis	Ma C.-S. et al. (2024)
Polyethylene glycol modification	Neutrophil	Thrombolytic agent tPA cross-linked with ROS-responsive polyethylene glycol and modified by insertion on the cell membrane surface	Polyethylene glycol: stable particle size, long circulation properties Cell membrane: biomimetic properties	Targeting thrombus sites and releasing tPA under the action of high concentrations of ROS locally, demonstrating thrombolytic and anti-inflammatory effects	Quan et al. (2024)
Glycosylation modification	Platelet	Salivary acid	Salivary acid: Unique binding affinity for L-selectin, which is abundantly expressed in circulating neutrophils Platelet membrane: Crossing the BBB into the damaged brain parenchyma	Attenuating neutrophil-induced brain damage and mitigating oxidative stress injury through effective scavenging of ROS	Song et al. (2024)
Membrane functionalization	Astrocyte membrane	CXCR3^+^	Bionic camouflage, immune clearance evasion and isotype targeting	Promoting protective autophagy in astrocytes, ameliorating cellular oxidative stress, and decreasing endothelin gene expression levels, thereby reducing secondary damage to neurovascular units	Liu Z. et al. (2024)
	Mesenchymal stem cell membrane	CXCR4^+^,RVG29 transmembrane peptides	Lesion homing, adsorption and neutralization of CXCL12	Ameliorating the mortality, reducing the infarct volume, and protecting neurogenic functions of neurons	Shi et al. (2022)

By integrating comprehensive biological properties and functionalities, multifunctional nanomaterials can significantly enhance the *in vivo* performance of therapeutic BMNPs. However, utilizing only a single type or conventional biomembrane coating often constrains the functional diversity of BMNPs. To address this limitation, researchers are increasingly exploring the use of hybrid biomembranes to prepare multifunctional biocomposite delivery systems. This approach involves combining biomembranes from different sources with nanomaterials to confer specific targeting and therapeutic capabilities, thereby expanding the functional scope of BMNPs. In addition, surface modification of BMNPs represents another strategy to enhance their targeting precisioncan (Jiménez-Jiménez et al., 2020). Following fusion between the biomembrane and nanocarrier, specific ligands—such as antibodies, DNA/RNA, peptides, proteins, and enzymes—are conjugated onto the membrane coating surface. This ligand functionalization enables specific binding to receptors on target cells, thereby achieving active targeting and enhancing specificity toward particular cells or tissues. Stroke homing peptides have emerged as a research focus in ischemic stroke therapy in recent years. By recognizing receptors highly expressed in ischemic brain regions, SHp can actively enrich at lesion sites. These peptides have been increasingly applied in preclinical studies of BMNPs for treating CIS.

Polyethylene glycol exhibits excellent hydrophilicity and flexibility, serving as a core strategy for nanodrug functionalization by prolonging circulation time, enhancing stability, enabling precise controlled release, and reducing toxicity. Deng Lang et al. utilized polyethylene glycol’s stable particle size and long-circulating properties to prepare polyethylene glycol-modified liposomes fused with pancreatic cancer PANC-1 cell membranes. Their study revealed that the cellular uptake of these nanoparticles by pancreatic cancer cells is simultaneously influenced by both polyethylene glycol-modified and cell membrane components (Deng et al., 2021). Furthermore, polyethylene glycol modification provides additional chemical groups and reactive sites on nanoparticle surfaces, facilitating further functionalization with targeting ligands for precise drug delivery. By crosslinking the thrombolytic agent tPA with ROS-responsive PEG and subsequently anchoring it onto neutrophil membranes via membrane insertion, a neutrophil-mimetic upconversion photosynthetic nanosystem was engineered. Guided by the neutrophil membrane, this system effectively targets thrombotic sites, demonstrating synergistic thrombolytic and anti-inflammatory effects (Quan et al., 2024).

Glycosylation modification in nanoscale formulations can significantly enhance their delivery efficiency and therapeutic efficacy by altering the physicochemical properties and biological interactions of the nanoparticle surface. Sialic acid is an important sugar molecule that is commonly used to decorate nanoparticles. Leveraging its unique binding affinity for L-selectin, which is abundantly expressed in circulating neutrophils, Junyan Song et al. developed a sialic acid-functionalized platelet-membrane-coated hollow Prussian blue nanoparticle loaded with deoxyribonuclease I. This neutrophil-hitchhiking system effectively traverses the BBB and accumulates in the injured brain parenchyma (Song et al., 2024). The severe toxic side effects of doxorubicin on the cardiovascular system, bone marrow, liver, and kidney functions have limited its clinical application. To address this, Wei Li et al.synthesized an amphiphilic nanomicelle by chemically conjugating lactose with doxorubicin, significantly enhancing the drug-loading capacity. Subsequently, they coated the nanomicelles with 4T1 mouse breast cancer cell membranes, which markedly improved biosafety and enhanced antitumor efficacy (Li W. et al., 2021).

Additionally, functional modification of the cell membrane of living cells to achieve high/low specific expression of membrane proteins is carried out to prepare BMNPs for *in vivo* targeting (Liu et al., 2019). Currently, the commonly studied surface modification methods primarily include:lipid insertion, chemical covalent modification, non-covalent adsorption, genetic modification, and metabolic engineering. Lipid insertion leverages the innate affinity of lipid molecules for cell membranes to anchor ligands onto nanoparticle surfaces. Chemical covalent modification utilizes reactive functional groups, including amines, thiols, and vicinal diols on membrane proteins or glycans to enable site-specific conjugation. Non-covalent adsorption predominantly achieves coupling through hydrophobic interactions such as fatty chain intercalation. Genetic modification facilitates targeted expression and surface display of functional membrane proteins, while metabolic glycoengineering modifies cellular surface carbohydrates to incorporate bioorthogonal functional groups for efficient, non-destructive bioconjugation (Li et al., 2024). Efficient drug delivery to the brain remains a paramount challenge in developing effective therapies for brain diseases (Moslemi et al., 2019). Attaining therapeutic efficacy typically requires elevated drug doses, which may precipitate dose-limiting toxicities. Emerging evidence demonstrates that intranasal administration circumvents the BBB, enabling targeted drug enrichment in cerebral tissues. As a non-invasive delivery modality, the nasal route facilitates direct transport of exogenous therapeutics from the nasal to the brain parenchyma via olfactory/trigeminal neural pathways (Agrawal et al., 2018). Therefore, in future research, it is worth considering the preparation of BMNPs suitable for nasal administration for the treatment of CIS.

The uncontrolled release of pharmaceuticals can significantly narrow the therapeutic window, underscoring the critical need for precise modulation of drug delivery to prevent both toxicity and therapeutic failure. Nanomedicine-based systems are designed to enable spatiotemporally controlled drug release, leveraging specific microenvironmental cues—such as elevated ROS levels or acidic pH—to achieve targeted therapeutic effects while minimizing off-target exposure. This adaptive release mechanism ensures that drugs are activated preferentially in pathological sites, optimizing treatment efficacy while reducing systemic adverse reactions. The multi-layered encapsulation of nanocarriers, biomembranes, and modifiers makes the release mechanism of the core drug in the body relatively complex. Once entering the body and facing the complex internal environment, the biomembrane of BMNPs will gradually degrade, slowly releasing the encapsulated drug. BMNPs primarily enter cells via endocytic internalization pathways. Surface properties of nanomedicines, such as positive charge or the presence of ligands that target cell surfaces, can significantly enhance cellular internalization and subsequent drug release. BMNPs exhibit complex *in vivo* release kinetics influenced by multiple factors, with the composition and structural heterogeneity of biomembranes critically regulating their physiological stability and degradation rates. Notably, red blood cell-derived membranes demonstrate extended stability through slow degradation, while tumor cell membranes rich in glycoproteins and glycolipids degrade more rapidly due to inherent proteolytic susceptibility (Gu Y. et al., 2024). Interestingly, many researchers are currently aiming to construct nanoscale drug delivery systems that respond to specific environments (pH, ROS) or stimuli (light, magnetism, temperature) to achieve precise lesion-specific drug release. However, subsequent investigations must continually address potential inflammatory responses triggered by degradation products from rapidly metabolized membrane materials in peri-lesional tissues. Optimizing cell membrane sources and designing disease-adapted delivery platforms to enhance spatiotemporal control over drug release kinetics thus remains critical for clinical translation (Rhaman et al., 2022). Furthermore, comprehensive toxicological assessment of BMNPs at advanced biological levels represents an essential path toward resolving toxicity barriers in clinical adaptation.

### 6.3 Clinical translation prospects

Currently, nanostructured drug delivery systems (e.g., liposomes) have achieved extensive clinical application (Krauss et al., 2019). Biologics of biological origin are also emerging in clinical practice, exemplified by stem cell therapies for Parkinson’s disease whose efficacy and safety have been validated in clinical trials (Sawamoto et al., 2025). While BMNPs are still in their infancy, and it is challenging to transform them into clinical trials. Although most BMNPs have completed the validation of efficacy and safety *in vitro* and in small animal models (mice, rats), they have not yet entered systematic toxicology studies in compliance with Good Laboratory Practice standards or the stage of Investigational New Drug application.

Conventional nanocarriers (e.g., liposomes) exhibit high batch-to-batch stability and standardized manufacturing, supported by well-established toxicity databases. Multiple polyethylene glycolylated/targeted liposomal formulations have advanced to Phase III trials or market approval. In contrast, BMNPs face significant clinical translation challenges: Inherent batch variability in biomembrane extraction and complex fabrication processes hinder compliance with Good Laboratory Practice equirements for formulation homogeneity; Critical data gaps in long-term toxicity profiles, including immunogenicity risks from residual membrane proteins and inconsistent cross-species toxicological correlations; Regulatory ambiguities regarding classification of these hybrid systems, given their combined nanostructured materials and biological components; Absence of standardized safety assessment frameworks specifically tailored for BMNPs. Notwithstanding these barriers, the innate advantages and therapeutic potential of BMNPs warrant continued research investment to bridge the lab-to-clinic gap.

## 7 Conclusions and outlook

Biomedical research now actively leverages advances in BMNPs, with biomimetic membrane nanotechnology demonstrating significant potential for CIS theranostics. By emulating biological membrane architecture and function, BMNPs enhance drug targeting specificity, reduce immunogenicity, and improve biocompatibility. BMNPs exhibit prolonged systemic circulation by evading reticuloendothelial system clearance, while substantially improving therapeutic efficacy in CIS through enhanced BBB penetration and reduced enzymatic drug degradation. Additionally, biomimetic membrane nanotechnology enables stroke diagnosis via biomarker-specific detection, demonstrating unique advantages for early pathological identification. This review systematically synthesizes contemporary BMNP preparation methodologies, characterization techniques, and applications of diverse membrane-camouflaged nanoagents in the diagnosis and treatment of CIS.

Despite the significant progress in the diagnosis and treatment of CIS with BMNPs, substantial challenges persist in the translation to clinical practice. First and foremost, optimizing the biocompatibility and stability of BMNPs is crucial for ensuring their safety and efficacy in clinical applications, making it a key research priority. The detailed molecular mechanisms underlying the *in vivo* performance of BMNPs and strategies to maximize therapeutic outcomes through precise spatiotemporal control of drug release also warrant in-depth investigation. BMNPs advance beyond simplified cellular/bacterial delivery systems by constructing immunocompetent nanoscale targeting platforms. However, this approach introduces biosafety challenges including antigenic responses to bacterial membrane components and potential infection risks requiring thorough investigation. Current research focuses on novel BMNPs designs to address these limitations—enhancing material-membrane compatibility for improved carrier stability, engineering multifunctional carriers for prolonged blood circulation, and developing tissue-specific nanoparticles to increase drug retention. These innovations are expected to drive the next-generation of advanced BMNPs therapeutics.

Large-scale clinical trials remain imperative to validate the efficacy and safety profiles of BMNPs.Future research should also focus on advance personalized CIS therapy through biomimetic membrane nanotechnology while integrating modern imaging modalities with genomic analytics to enhance diagnostic precision and treatment personalization. As nanotechnology and biomedicine progressively converge, biomimetic membrane nanotechnology is poised to assume increasingly pivotal theragnostic roles in CIS management. Future research should establish standardized frameworks for BMNPs in CIS injury, concurrently advancing innovative nanotechnology to develop clinically translatable platforms. This translation will expedite conversion of nano-delivery therapeutic advantages into tangible health outcomes for CIS patients. Notwithstanding enhanced drug targeting efficacy, BMNPs face persistent safety challenges including immunogenicity risks, bioactive component instability, and imprecise spatiotemporal drug release control—issues demanding comprehensive resolution in subsequent investigations.
