# Correction: Biomimetic membrane nanotechnology in cerebral ischemic stroke: a promising technology for therapeutic treatment and diagnosis

**DOI:** 10.3389/fbioe.2025.1729877

**Published:** 2025-11-07

**Authors:** Yu Long, Zhiyan Zou, Yuanyuan Wu, Huiyi Feng, Ting Chen, Zhi Yang, Xuemin Jian, Yuan Yin, Xiaoan Li

**Affiliations:** 1 NHC Key Laboratory of Nuclear Technology Medical Transformation, Mianyang Central Hospital, School of Medicine, University of Electronic Science and Technology of China, Mianyang, China; 2 State Key Laboratory of Southwestern Chinese Medicine Resources, School of Pharmacy, Chengdu University of Traditional Chinese Medicine, Chengdu, China; 3 School of Animal Science, Xichang University, Xichang, China; 4 Department of Gastroenterology, National Clinical Key Specialty, Mianyang Central Hospital, School of Medicine, University of Electronic Science and Technology of China, Mianyang, China

**Keywords:** biomimetic membrane nanopreparations, cerebral ischemic stroke, therapy, diagnostics, development prospects

The funder “Mianyang Science and Technology Bureau, Grant Number: 2023ZYDF073” was erroneously omitted. The correct funding statement reads:

The author(s) declare that financial support was received for the research and/or publication of this article. This work was supported by Sichuan Natural Science Foundation (Number: 2025ZNSFSC1801, 2025ZNSFSC1727), Mianyang Central Hospital Talent Introduction Research Project (Number: 2025RCYJ-003), NHC Key Laboratory of Nuclear Technology Medical Transformation (MIANYANG CENTRAL HOSPITAL) (Number: 2024HYX020), Mianyang Central Hospital Incubation Subjects (Number: 2024FH006), Sichuan Science and Technology Program (Number: 2023YFS0470). Liangshan Prefecture Science and Technology Bureau project (Number: 24DXWT0001). Mianyang Science and Technology Bureau (Number: 2023ZYDF073).

The original article has been updated.

