# Supplementary material for: Biomimetic membrane nanotechnology in cerebral ischemic stroke:a promising technology for therapeutic treatment and diagnosis
Source: Front Bioeng Biotechnol. 2025 Aug 20;13:1605377. doi: 10.3389/fbioe.2025.1605377 (PMC12405248; doi:10.3389/fbioe.2025.1605377)
Supplement: Supplementary file 1 [file Table1.docx]

**Table 2** The therapeutic CIS effects of BMNPs prepared from cell membrane

| **Source** | **Drug carrier** | **Nanocarrier** | **Modification** | **Cell membrane action characteristics** | **Pharmacological effect** | **Reference** |
| --- | --- | --- | --- | --- | --- | --- |
| Red blood cell  membrane | Rapamycin | Sulfated chitosan polymer core | SHp | Prolonging retention time in the body | Promoting microglia polarization, maintaining the integrity of the BBB, reducing cerebral infarction, and promoting cerebral neurovascular remodeling | Cao et al. (2024) |
| Red blood cell  membrane | NR2B9C | Dextran polymer core | SHp | Prolonging retention time in the body | Great protection against glutamate-induced PC-12 cytotoxicity, prolonging the somatic circulation of NR2B9C, enhancing active targeting of ischemic regions and reducing ischemic brain damage in MCAO rats | Lv et al. (2018) |
| Platelet membrane | Deoxyribonuclease I | Nanoenzymes | Salivary acid | Crossing the BBB into the damaged brain parenchyma | Attenuating neutrophil-induced brain damage and mitigating oxidative stress injury through effective scavenging of ROS | Song et al. (2024) |
| Platelet membrane | tPA | Liposome | Annexin V | Targeting thrombus properties | Improving the ability of tPA to target thrombus sites and anti-platelet activation, improving neurological function, increasing focal cerebral perfusion, reducing infarct size, and decreasing blood-brain barrier permeability | Quan et al.  (2022) |
| Platelet membrane | L-arginine and γ-Fe2O3 | Magnetic Nanocarriers |  | Targeting ischemic stroke lesions, locating ischemic tissue and vascular plaque sites | Dissolving blood clots and dilating blood vessels to extend the golden window of time for treatment of ischemic stroke | Li et al. (2020) |
| Platelet membrane | Zein, docosahexaenoic acid, recombinant human tissue plasminogen activator | Cu_4.6_O nanoparticle |  | Targeting damage to blood vessels and blood clots | Excellent thrombolytic effect, promoting microglia polarization and restoring neurobiological and behavioral functions | Kong et al. (2024) |
| Platelet membrane | Melanin, tPA | Nanoparticle |  | Targeting thrombus properties | Significantly enhanced therapeutic efficacy in ischemic stroke with cascade thrombus targeting, precise thrombolysis, and ischemia-reperfusion protection characteristics | Yu et al.  (2022) |
| Fusion of Platele membranes with microglia membrane | Sphingosinekinase 1 siRNA | Nanocomplex |  | Platelet membrane: targeting of thrombotic properties. Microglia membrane: isotype targeting, CD29-mediated recognition of CD106, triggering receptor-mediated endocytosis, highly efficient uptake by microglia. | Breaking through the in vivo delivery barrier of siSPHK-1 and realizing gene silencing in microglia; improving the inflammatory microenvironment at the site of cerebral ischemia/reperfusion injury | Wu (2023) |
| 4T1 cell membrane | Succinobucol | pH-sensitive polymeric nanovehicle |  | Preferential targeting of ischemic brain hemispheres, | Significantly enhancing ischemic cerebral microvascular reperfusion, resulting in a 69.9% reduction in infarct volume, significant neuroprotection, superior to the corresponding non-camouflaged nanocarriers | He et al. (2021) |
| Fusion of 4T1 tumor cell membranes with platelet membranes | Paeonol and polymetformin | Liposome |  | 4T1 tumor cells: BBB penetration.  Platelets: targeting injured vascular system | Efficiently targeting ischemic lesion, preventing neuroinflammation, scavenging excess ROS, reprogramming microglia phenotypes, and promoting angiogenesis | Tang et al. (2024) |
| Neutrophil-like cell m embrane |  | Mesoporous Prussian blue nanozyme |  | Targeting and homing of lesions, binding to inflamed brain microvascular endothelial cells | Promoting microglia polarization to M2, reducing neutrophil recruitment, neuronal apoptosis, and proliferation of neural stem cells, neuronal precursors, and neuronal | Feng et al. (2021) |
| Differentiated HL-60 cells membrane | Fingolimod hydrochloride | Nanoparticle |  | Crossing the BBB to the site of brain injury | Delivering 15.2-fold more Fingolimod hydrochloride into the ischemic brain, reducing the risk of cardiotoxicity and infection, reprogramming microglia toward anti-inflammatory phenotypes | Zhao et al. (2024) |
| Neutrophil membrane | Leonurine | Nanoliposomal |  | Penetrating BBB, targeting infarct core, neutralizing inflammation, immuno-avoidance properties | Alleviating neuronal apoptosis, oxidative stress, neuroinflammation, and restoring BBB integrity in transient middle cerebral artery occlusion (tMCAO) rats | Tang et al. (2023) |
| Neutrophil membrane | Cannabidiol | Nanoparticle | α-Lipoic | Converging to the infarct core and affinity for inflammatory cytokines | Reducing deleterious factors and infarct volume in the core and semidarktic band, improving neurologic recovery | Liu et al. (2022) |
| Neutrophil membrane | Edaravone | Nanoparticles | SHp | Targeting Inflammation | Precise Targeting of CIR sites, scavenging the ROS, inhibiting inflammation, and providing neuroprotection | Dong Z.  et al. (2024) |
| Macrophage membrane | phosphorous dendrimer (termed as AK137)/fibronectin，edaravone | Nanocomplexes |  | Crossing BBB | Alleviating oxidative stress, polarizing microglia M2, decreasing pro-inflammatory cytokine secretion, and acting on neuronal cells to fight apoptosis | Ma J. et al. (2024) |
| Macrophage membrane | Apelin-13 | Nanoparticle | Distearoyl phosphatidylethanolamine-polyethylene glycol-RVG29 | Crossing the BBB and selectively accumulating in ischemic and inflamed areas | Improving neurological scores and reducing infarct volume, inhibiting NLRP3 inflammasome-mediated pyroptosis | Ma C-S. et al. (2024) |
| Macrophage membrane | Ginsenoside Rg3 and Panax notoginseng saponins | Liposome |  | Crossing the BBB, affinity for inflamed cerebral microvascular endothelial cells, specific targeting of ischemic sites | Exhibiting stronger brain targeting and prolonging drug half-life | Liu T. et al. (2024) |
| Macrophage membrane | Fingolimod | Honeycomb manganese dioxide (MnO2) nanosphere |  | Mediating recognition with cell adhesion molecules | Reducing oxidative stress and promote the transition of M1 microglia to M2 type, reversing the proinflammatory microenvironment and reinforcing the survival of damaged neuron | Li C. et al. (2021) |
| Macrophage membrane protein | lncRNA-EPS | Liposome |  | Immune evasion | Targeting inflammatory cells and accelerated neuron regeneration by promoting inflammation resolution | Zhang et al. (2020) |
| M2-macrophage membrane | Baicalin | Poly lactic-co-glycolic acid nanoparticles |  | Efficiently and actively targeting ischemic brain tissue and accumulating in microglia and neurons | Reprogramming microglia from M1 to M2, reducing neutrophil infiltration and inhibiting neuronal apoptosis | Zhang et al. (2025) |
| Macrophage membrane | Angelica polysaccharides, Resveratrol | Nanoparticle |  | Crossing BBB | Improved the area of cerebral infarction and improved neurological function scores | Su et al. (2022 a) |
| Macrophage membrane | Angelica polysaccharide、Ethyl ferulate, Tetramethylpyrazine | Nanoparticle |  | Crossing BBB | Effectively delivering drugs to the site of brain injury, significantly reducing the infarct size | Su et al. (2022 b) |
| Macrophage membrane | Baicalin | Liposome |  | Enhancing brain targeting and improving circulation of Baicalin in the bloodstream | Significantly improving neurological deficits, cerebral infarct volume and cerebral pathology in MCAO rats | Long et al. (2022) |
| Monocyte membranes | Rapamycin | Nanoparticles |  | Actively targeting and binding to inflammatory endothelial cells, thereby inhibiting monocyte adhesion to endothelial cells | Ameliorating nerve scores and infarct volume | Wang et al. (2021) |
| Microglia membrane membrane | anti-Repulsive Guidance Molecule a monoclonal antibody，Fe3O4，perfluorohexane | Liposome |  | Targeting the ischemia-damaged endothelial cells | Protecting the ischemic region from ischemia/reperfusion injury allows visualization of thrombus by ultrasound/photo-acoustic imaging | Cheng et al. (2024) |
| M2 - microglial cell membrane | Polyphenol tannic acid, poloxamer 188, catalase | Nanoparticle |  | Intrinsic ischemia-homing and BBB-crossing capabilities | Integrating multiple therapeutic mechanisms to synergistically reduce inflammation and protect neurons | Duan et al. (2022) |
| M2 - microglial cell membrane | PEG-SMA-F11, P123 | Nano Oxygen Delivery System |  | High affinity for brain inflammatory vascular endothelial cells | Increasing the partial pressure of oxygen at the lesion in the early stage of ischemia, reducing apoptosis, inhibiting MMP-9 secretion, protecting the integrity of the BBB, and reducing cerebral edema | He et al. (2023) |
| CXCR3^+^ astrocyte membrane | Rapamycin and si EDN1 | lipid vesicle | RVG29 transmembrane peptides | Bionic camouflage, immune clearance evasion and isotype targeting | Promoting protective autophagy in astrocytes, ameliorating cellular oxidative stress, and decreasing endothelin gene expression levels, thereby reducing secondary damage to neurovascular units | Liu Z. et al. (2024) |
| Mesenchymal stem cell membrane | ZL006 | Prussian blue nanoparticles |  | Reducing the immunogenicity, enhancing homing ability to the cerebral ischemic penumbra | Selectively targeting the cerebral ischemic penumbra to reduce infarct size, protect neurogenic function, and improve mortality | Zhang et al. (2024) |
| Mesenchymal stem cell membrane | Dl-3-n-butylphthalide | Liposome |  | Enhancing the ability to accurately target the injured hemisphere | Promoting recovery of motor function in model mice by improving the damaged microenvironment and suppressing neuroinflammation | Dong Y-F. et al. (2024) |
| CXCR4^+^ mesenchymal stem cell membrane | A151 (cGAS inhibitor, telomerase repeat sequences) | Nanoparticle |  | Improving the homing of nanoparticles to the cerebral ischemic lesions, adsorbing and neutralizing CXCL12 to cut off infiltration of peripheral-neutrophils and mononuclear macrophages | Ameliorating the mortality, reducing the infarct volume, and protecting neurogenic functions of neurons | Shi et al. (2022) |
| Mesenchymal stem cell membrane | Curcumin | Liposome |  | Targeting, long-circulating capacity, immune masking and multiligand integration properties | Improving survival in mice with ischemic stroke | Wu et al. (2020) |
